# The structure of the Milky Way from period-luminosity relations

**DOI:** 10.1017/S1743921323003083

**Published:** 2024-02-06

**Authors:** Dorota M. Skowron

**Affiliations:** Astronomical Observatory, University of Warsaw, Aleje Ujazdowskie 4, 00-478 Warsaw, Poland

**Keywords:** Classical Cepheids, RR Lyrae, Delta Scuti, Miras, Long Period Variables, Milky Way Structure

## Abstract

Disentangling the structural components of the Milky Way requires knowledge of distances to various classes of objects, both young, which trace the Galactic disk, and old, which trace the Galactic bulge and halo. Variable stars that obey period-luminosity relations are perfect distance indicators for such studies. Here we discuss recent findings on the structure of our galaxy, inferred from period-luminosity relations for both young, old and intermediate-age variable stars, including Cepheids, RR Lyrae stars, *δ* Scuti stars, and long-period variables.

## 1 Introduction

All of us astronomers would like to know what our own galaxy, the Milky Way, looks like. And we often see the artist impressions of the Milky Way disk showing the bright central overdensity and spiral arms full of star formation and prominent dust lanes. These are of course based on real data and models, and backed up by photographs of other galaxies in the Universe, but are nevertheless only schematic visualizations of what our galaxy might look like.

However, we agree by now, that we live in a spiral galaxy, with three main components: the bulge, the disk, and the halo. And in order to be able to constrain the structure of the Milky Way, we need to have tracers in each of these components, to which we can calculate reliable distances that will make it possible to investigate their three-dimensional distribution and thus the shape of our galaxy.

Fortunately, there are several groups of pulsating variable stars in the classical instability strips on the Hertzsprung-Russel diagram (see, e.g., Figure 1 in Kurtz 2022), both old and young, that can serve as tracers of the Galactic structure. The oldest group, i.e. the RR Lyrae stars, are ideal for learning about the bulge and the halo of the Galaxy. The youngest, i.e. the Classical Cepheids, have been successfully used to study the structure of the Milky Way disk. And finally, the intermediate group, including Type II Cepheids, *δ* Scuti, and Mira-type stars (which are in fact a mixture of the old, young, and intermediate populations), located all-sky, can help us understand each of the components of our galaxy.

**Figure 1:**
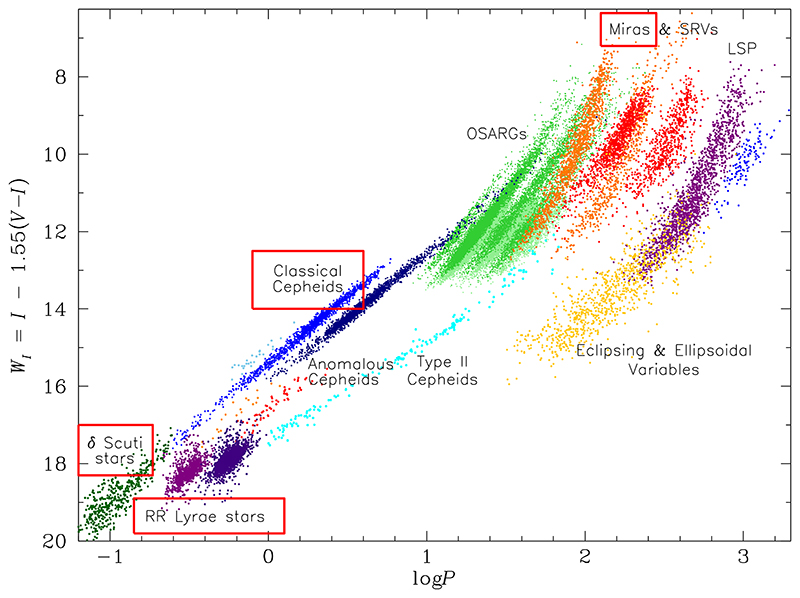
All-in-one period-Wesenheit relation for pulsating variable stars discovered by the OGLE survey in the Large Magellanic Cloud. The period *P* is in days and the Wesenheit index *W_I_* is in magnitudes. Red frames mark variables that have been used to study the structure of the Milky Way galaxy. The top right part of the plot is occupied by long-period variable stars which are further subdivided into Miras, semi-regular variables (SRVs), OGLE small-amplitude red giants (OSARGs), long secondary period variables (LSP), eclipsing, and ellipsoidal variables. The plot was provided by Igor Soszyński.

Figure 1 shows the all-in-one period-luminosity diagram, or the Leavitt law, for all known classes of pulsating variable stars in the Large Magellanic Cloud (courtesy of Igor Soszyński), observed by the Optical Gravitational Lensing Experiment (OGLE) survey (Udalski *et al*. 2015). The fact that all these stars are located roughly at the same distance and that the reddening-free Wesenheit index was used as a measure of brightness allows us to construct such tight period-luminosity relations.

In the case of our galaxy, stars are at different distances from us, so constructing periodluminosity relations is possible only for a subset of objects with distances known from other methods. Then, after calibrating the period-luminosity relation, it can be used to calculate distances to other stars of the same variable type, and as a result, to study the Galactic structure populated by this group of stars.

So far, four groups of pulsating variable stars in the Milky Was have been used to study its structure, and these groups are marked with red frames in Figure 1. They include RR Lyrae-type stars, Classical Cepheids, *δ* Scuti stars, and Miras.

## 2 Pulsating variable stars in numbers

During this Symposium Igor Soszyński told us about the revolution in the variable star research by the OGLE survey, which started in the very beginning of this century and continues until now, with over 1,088,000 variable stars classified so far. Gisella Clementini spoke about another revolution, by the *Gaia* mission, which constitutes over 9,976,000 variable stars released in the Data Release 3 (DR3), of which the majority are new discoveries. These numbers are overwhelming and provide an excellent database for numerous astrophysical studies. And while the majority of discoveries had been made by *Gaia* and OGLE, there are of course other important surveys that significantly contributed to this research area by providing data for the four variable star groups that have been used to study the Galactic structure. In an attempt to gather all these contributions in one place, we reference appropriate catalogs below, although the list is most certainly not complete, for which we apologize the reader.

### 2.1 RR Lyrae-type stars

The current number of all-sky RR Lyrae stars is around ~271,000, of which about ~223,900 are members of the Milky Way and the remaining ones belong mostly to the Magellanic Clouds. The main contributions to the RR Lyrae sample in our galaxy are from OGLE (Soszyński *et al*. 2011a, 2014, 2019) and *Gaia* (Clementini *et al*. 2023), but also from the All Sky Automated Survey (ASAS; Pojmanski 2002, 2003; Pojmanski & Maciejewski 2004, 2005; Pojmanski, Pilecki, & Szczygiel 2005), the All Sky Automated Survey for SuperNovae (ASAS-SN; Jayasinghe *et al*. 2019), the Catalina Sky Survey (CSS; Drake *et al*. 2013, 2014; Torrealba *et al*. 2015; Drake *et al*. 2017), the Lincoln Near-Earth Asteroid Research project (LINEAR; Palaversa *et al*. 2013; Sesar *et al*. 2013), the Panoramic Survey Telescope and Rapid Response System (Pan-STARRS1; Sesar *et al*. 2017), the Palomar Transient Factory (PTF; Sesar *et al*. 2014), the VISTA Variables in the Via Lactea survey (VVV; Contreras Ramos *et al*. 2018; Dékány *et al*. 2018), and the Zwicky Transient Facility (ZTF; Chen *et al*. 2020), as well as many other sources compiled in the General Catalog of Variable Stars (GCVS; Samus’ *et al*. 2017).

**Figure 2:**
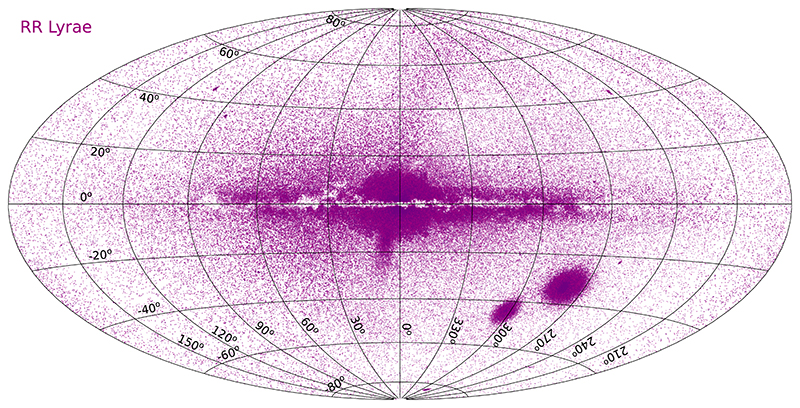
The all-sky distribution of RR Lyrae-type variable stars in Galactic coordinates.

Figure 2 shows the on-sky distribution of RR Lyrae-type variables from these catalogs.

### 2.2 Classical Cepheids

**Figure 3:**
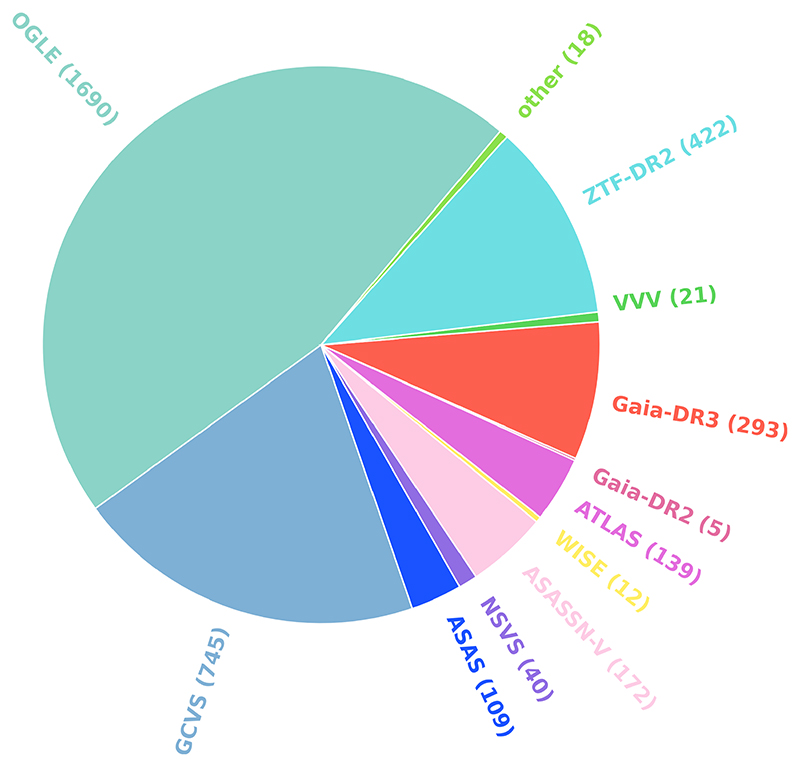
The number of Classical Cepheids by discovery survey and GCVS.

The current number of known Classical Cepheids in the Milky Way is 3,666, with the main contributions from OGLE (Pietrukowicz *et al*. 2013; Soszyński *et al*. 2017; Udalski *et al*. 2018; Pietrukowicz, Soszyński, & Udalski 2021) and from other surveys, as pictured in Figure 3, including ASAS (Pojmanski 2002, 2003; Pojmanski & Maciejewski 2004, 2005; Pojmanski, Pilecki, & Szczygiel 2005), ASAS-SN (Jayasinghe *et al*. 2019), the Asteroid Terrestrial-impact Last Alert System (ATLAS; Heinze *et al*. 2018), *Gaia* (Ripepi *et al*. 2023), and ZTF (Chen *et al*. 2020), as well as many other sources compiled in the GCVS (Samus’ *et al*. 2017).

**Figure 4:**
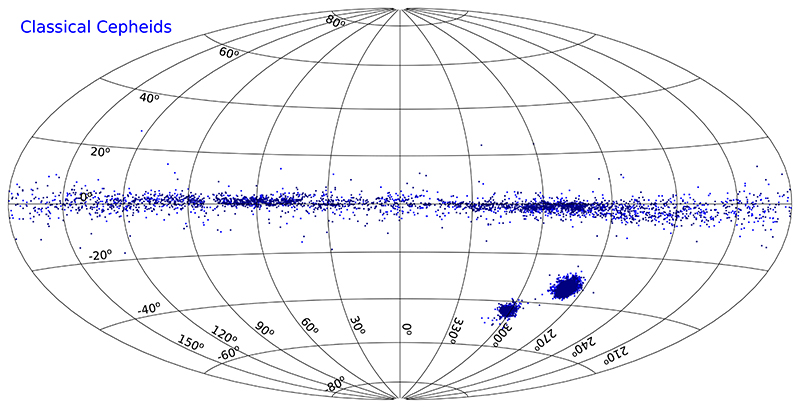
The all-sky distribution of Classical Cepheids in Galactic coordinates.

There are a number of Cepheids discovered by infrared (IR) surveys, e.g., VVV (Dékány *et al*. 2019), but here only optically confirmed Cepheids are included, because nearly sinusoidal light curves of Cepheids in the IR makes their classification unambiguous. Figure 4 shows the on-sky distribution of Classical Cepheids from these catalogs.

The up-to-date list of all genuine Galactic Classical Cepheids† is maintained by Paweł Pietrukowicz (Pietrukowicz, Soszyński, & Udalski 2021) and is being regularly updated as new discoveries are made.

### 2.3 Mira-type stars

Catalogs of Miras in the Milky Way are not as complete as in the case of RR Lyrae stars or Classical Cepheids. Nevertheless, recent years have seen much progress and there are currently at least ~76,000 known Galactic Mira type stars. It is difficult to establish a more precise number, because some surveys only provide a “long-period variable” classification, without further subdivision into Miras, SRVs, OSARGs, etc. Catalogs of Miras have been published by OGLE (Soszyński *et al*. 2013; Iwanek *et al*. 2022), ASAS (Pojmanski 2002, 2003; Pojmanski & Maciejewski 2004, 2005; Pojmanski, Pilecki, & Szczygiel 2005), ASAS-SN (Jayasinghe *et al*. 2019), ATLAS (Heinze *et al*. 2018), CSS (Drake *et al*. 2014, 2017), *Gaia* (Lebzelter *et al*. 2023), VVV (Nikzat *et al*. 2022; Sanders *et al*. 2022), and ZTF (Chen *et al*. 2020), as well as many other sources compiled in GCVS (Samus’ *et al*. 2017).

**Figure 5:**
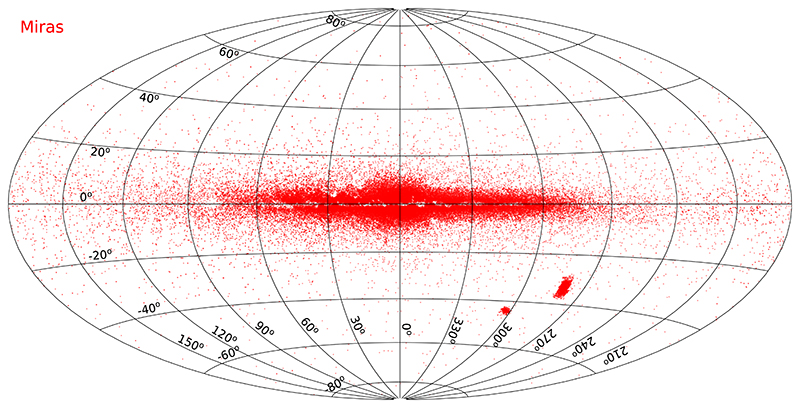
The all-sky distribution of Mira-type variable stars in Galactic coordinates.

Figure 5 shows the on-sky distribution of Mira-type variables from these catalogs.

### 2.4 *δ* Scuti-type stars

There were 636 known *δ* Scuti stars two decades ago (see compilation by Rodríguez, López-González, & López de Coca 2000). This number has greatly increased and we now know over 33,000 *δ* Scuti stars in our galaxy, the majority of which were discovered by the OGLE survey (Pietrukowicz *et al*. 2020a, 2022; Soszyński *et al*. 2021) but also by ASAS-SN (Jayasinghe *et al*. 2019) and ZTF (Chen *et al*. 2020).

**Figure 6:**
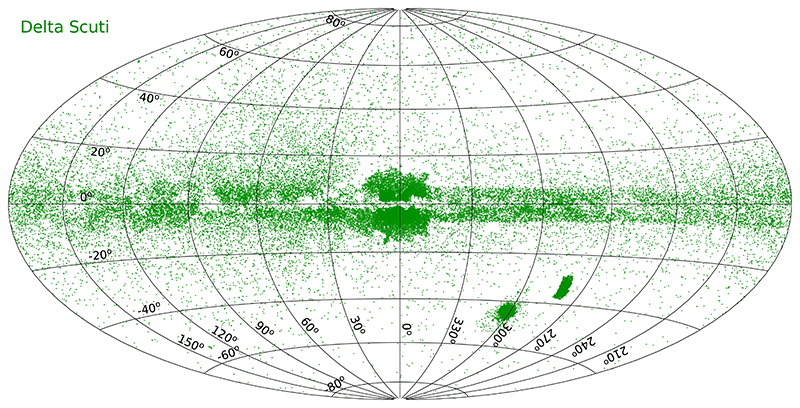
The all-sky distribution of *δ* Scuti-type variable stars in Galactic coordinates.

Figure 6 shows the on-sky distribution of *δ* Scuti-type variables from these catalogs.

## 3 The Milky Way structure

There are three main components of our galaxy: the bulge, the disk, and the halo. Vasily Belokurov gave an informative overview of the structure of the halo, so the focus here will be put on the remaining parts of the Milky Way.

### 3.1 The bulge

The bulge is composed mainly of old and intermediate-age stars, and thus most of the structural studies of the bulge, using pulsating variable stars, have been made with the use of RR Lyrae, Miras, and recently also *δ* Scuti-type stars.

#### 3.1.1 The bulge in RR Lyrae

The first study that used a large sample of bulge RR Lyrae stars was performed by Pietrukowicz *et al*. (2015) with over 27,000 objects from the OGLE-III survey. By modelling the RR Lyrae on-sky distribution and density profile, the authors found that the old bulge has the shape of a triaxial ellipsoid (see their Figures 6 and 8) with a major axis inclined to the line of sight by 20° within the Galactic plane. However, the X-shaped structure reported by studies of red clump giants in the bulge (McWilliam & Zoccali 2010) was not observed.

With the release of over 78,000 RR Lyrae in the bulge and disk area from OGLE-IV, Pietrukowicz *et al*. (2020b) investigated the properties of the old populations of the Milky Way’s bulge, disk, and halo using both period-luminosity relations and photometric metallicity estimations. Their Figure 2 shows the distribution of RR Lyrae in these three components of our galaxy, while their Figure 3 presents metallicity distributions for each component, with the distribution maxima at [Fe/H] = −1.2 dex, −1.0 dex, and −0.6 dex for the halo, bulge, and disk, respectively. From the shape of the metallicity distributions, the authors conclude that halo stars are present deep in the bulge region.

Further analysis shows that about 25% of bulge RR Lyrae belong to the halo population (see Figure 4 of Pietrukowicz *et al*. 2020b), which is in excellent agreement with spectroscopy-based results by Kunder *et al*. (2020) based on the BRAVA-RR data, who found that 25% of bulge RR Lyrae are halo interlopers (see their Figure 6), but is slightly more than the 9% reported by Prudil *et al*. (2019) based on a kinematical study of bulge RR Lyrae. Kunder *et al*. (2020) also showed that, once the halo interlopers are removed from the bulge sample, we can clearly see two populations of RR Lyrae that are spatially and kinematically different. The existence of two bulge populations was also found by Pietrukowicz *et al*. (2020b) – their analysis of the period-amplitude diagram of bulge RR Lyrae revealed two sequences with likely different metallicities (see their Figures 6 – 8). The presence of multiple old populations in the Milky Way bulge was also observed in IR data (Dékány *et al*. 2018) and suggests that the bulge has been formed through merger events.

#### 3.1.2 The bulge in Miras and *δ* Scuti

The old and intermediate-age bulge populations are represented by both Mira-type stars and *δ* Scuti-type stars, which have recently been used to study the Galactic bulge region.

A study of a clean sample of almost 7,500 *δ* Scuti stars from the OGLE catalogs is the first study of such a large sample of these variables in the bulge region (Deka, Deb, & Kurbah 2022). The authors determine the geometrical parameters of the bulge with the use of a fully Bayesian Markov Chain Monte Carlo analysis, and find that the *δ* Scuti distribution has the form of a triaxial ellipsoid (no X-shape morphology), where a bar-like structure is inclined at the angle of 22° to the line of sight, which is similar to results obtained by Pietrukowicz *et al*. (2015) for RR Lyrae stars.

The picture is not as consistent in the case of Mira-type variables. Both López-Corredoira (2017) and Chrobáková, López-Corredoira, & Garzón (2022) have shown that the population traced by these stars (with an average age estimated at ~ 9 Gyr) can be described as a boxy bulge, without the presence of an X-shaped structure, which is consistent with the results presented by Pietrukowicz *et al*. (2015) for RR Lyrae stars and Dékány *et al*. (2019) for Type II Cepheids. However, Catchpole *et al*. (2016) showed that the the bulge structure is a function of age, such that the younger Miras (age ≲ 5 Gyr) follow a bar-like structure inclined to the line of sight, which may be part of an X-shaped structure, while the older ones follow a more spheroidal distribution (see their Figure 11).

The dependence of bulge morphology on age and metallicity has also been recently investigated by Grady, Belokurov, & Evans (2020), who found that the older Miras (~ 9 – 10 Gyr) show little bar-like morphology, while the younger ones do (with a possible X-shaped structure), with the inclination angle of ~ 21° being similar to those of RR Lyrae, *δ* Scuti, and Type II Cepheids.

The most recent study of the largest by far sample of ~66,000 Miras in the Milky Way bulge and disk was done by Iwanek *et al*. (2023). The authors used a much more complex model containing three barred components, and found that the X-shaped structure is present in the bulge (see their Figures 4 – 6). The analysis, however, did not include the age dependence.

### 3.2 The disk

The disk of the Milky Way is composed of stars, gas, and dust, and is an environment where most of the star formation is happening. For this reason, the youngest stars that are born in the disk are excellent tracers of its structure, as they have not had enough time to move away from their birthplaces.

#### 3.2.1 The disk in Classical Cepheids

**Figure 7:**
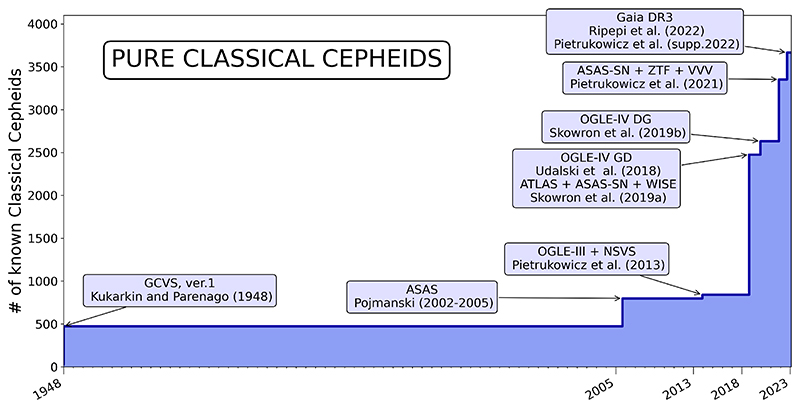
The change of the number of known Classical Cepheids in the Milky Way with time.

Classical Cepheids are typically younger than 300 – 400 Myr, very bright, and obey a period-luminosity relation, which makes them ideal to study the shape of the Galactic disk. Recent years have seen exponential growth in the number of known Galactic Classical Cepheids (Figure 7), and we can safely say that the majority of these stars in the Milky Way have been discovered by now. This has led to a number of publications that used Cepheids to study the disk morphology (Skowron *et al*. 2019a,b; Chen *et al*. 2019; Dékány *et al*. 2019; Minniti *et al*. 2021; Poggio *et al*. 2021; Lemasle *et al*. 2022; Gaia Collaboration *et al*. 2023).

All of these studies are similar in the sense that they are based on more or less the same set of stars, and use period-luminosity relations to determine Cepheid distances. At the same time they differ, because different period-luminosity and period-age relations (to select the youngest Cepheids) are used in each study. For example, Skowron *et al*. (2019a,b) utilize mid-IR data from *Spitzer* and WISE for Cepheids and appropriate period-luminosity relations in the mid-IR, and correct for extinction with IR extinction maps; Gaia Collaboration *et al*. (2023) make use of period-Wesenheit-metallicity relations for *Gaia* bands and do not correct for extinction; while Lemasle *et al*. (2022) use period-Wesenheit relations in mid-IR WISE bands also without the extinction correction.

**Figure 8:**
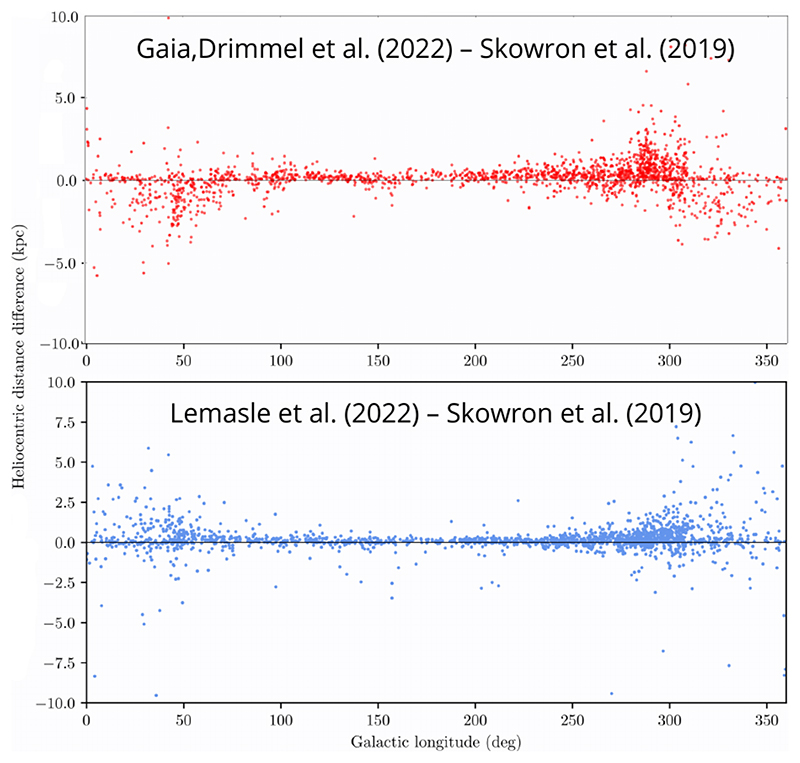
The difference between Classical Cepheid distances calculated with slightly different methods. Top: distance difference between the study of Gaia Collaboration *et al*. (2023) and Skowron *et al*. (2019a,b) vs. Galactic longitude. Bottom: distance difference between the study of Lemasle *et al*. (2022) and Skowron *et al*. (2019a,b) vs. Galactic longitude, provided by Bertrand Lemasle. The Galactic anti-center is at Galactic longitude 180°.

Thus, it is not surprising that Cepheid distances obtained in each study differ, which is illustrated in Figure 8, where the top panel shows how the distance difference between the study of Gaia Collaboration *et al*. (2023) and Skowron *et al*. (2019a,b) changes with Galactic longitude, while the bottom panel shows the same relation, but between the study of Lemasle *et al*. (2022) and Skowron *et al*. (2019a,b). Interestingly, the distance differences are largest toward the Galactic center, indicating that the treatment of extinction has a large impact on the final results. Using the reddening-free Wesenheit index, which by construction assumes a constant ratio of total-to-selective extinction, may not be appropriate in the highly extincted regions of the Milky Way, where the reddening law is not fully understood (Schlafly *et al*. 2016; Nataf *et al*. 2016). This is further supported by the fact that distances calculated with different methods are consistent toward the anti-center (around longitude 180°), where there is much less reddening.

**Figure 9:**
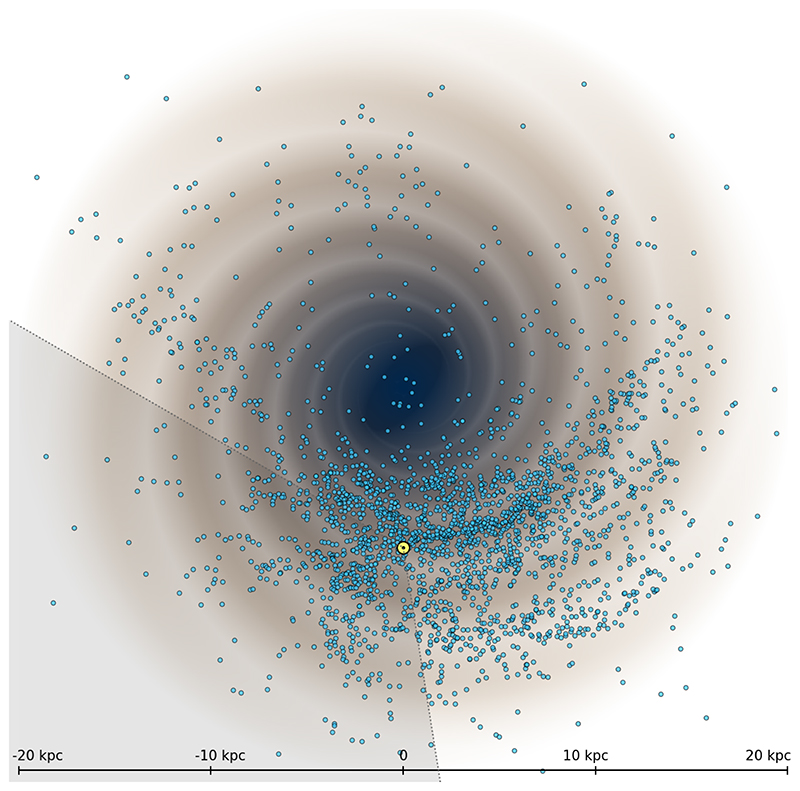
The top view of 2,390 Classical Cepheids in the Milky Way plotted over the spiral arm model consistent with the neutral hydrogen distribution (Levine, Blitz, & Heiles 2006). The image is based on data from Skowron *et al*. (2019a,b) and does not include Cepheids recently discovered from ASAS-SN, *Gaia*, and ZTF. The Sun is marked with a yellow dot. The gray area at the bottom left shows the region unavailable to the OGLE survey.

Nevertheless, the distribution of Galactic Classical Cepheids has been successfully used to study the structure of the Milky Way disk. Figure 9 shows the top view of Cepheids in our galaxy based on the work of Skowron *et al*. (2019a,b). We see clear signs of spiral structure, although less evident in the outer disk, where the median age of Cepheids is larger (compare with Figure 3 in Skowron *et al*. 2019b). This is expected, because older Cepheids (150 – 250 Myr) have had enough time to move away from the spiral arm that they were born in, and as a result could be in the neighboring arm or the inter-arm region.

**Figure 10:**
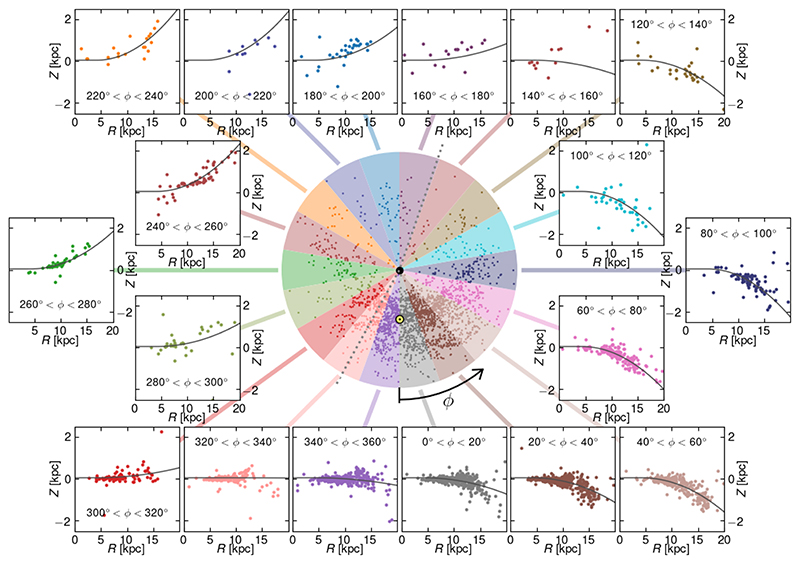
The model of the Galactic warp adapted from Skowron *et al*. (2019a). The central disk is divided into 18 colored regions in the Galactocentric polar coordinate system. The Sun is marked with a yellow dot while the Galactic center with a black dot. The azimuth *ϕ* = 0° is pointing toward the Sun, while the dotted line divides parts of the disk model warped South and North. The panels show the vertical distribution of Cepheids within each colored region, where the Galactocentric distance *R* is plotted against distance from the Galactic plane *Z*. Black lines show the intersection of the model surface fitted to the three-dimensional distribution of Cepheids.

It has been long known that the Milky Way disk observed in neutral hydrogen is warped. Figure 10 (based on Skowron *et al*. 2019a) shows the the top view of the Galaxy in the middle (analogous to that in Figure 9), with individual Cepheids marked with colored dots. The disk is divided into 18 colored regions, and then the vertical distribution of Cepheids within each region is shown in 18 side panels with matching colors. The panels show the Galactocentric distance *R* plotted against distance from the Galactic plane *Z*, and the black lines show the intersection of the model surface fitted to the three-dimensional distribution of Cepheids. The agreement between the model and the observed distribution of Classical Cepheids is surprisingly good, even in the less populated sectors on the far side of the Galactic center, i.e., 140° < *ϕ* < 160°, 160° < *ϕ* < 180°, and 200° < *ϕ* < 220°.

Figure 10 also shows that the Milky Way disk is flared, that is the farther from the Galactic center, the thicker the disk is. The observed degree of flaring matches the one seen in neutral hydrogen.

There have been various attempts (Minniti *et al*. 2021; Poggio *et al*. 2021; Gaia Collaboration *et al*. 2023; Lemasle *et al*. 2022) to model the spiral structure of the Milky Way using the distribution of Classical Cepheids, or to match their distribution to existing spiral arm models based on neutral hydrogen (e.g. Levine, Blitz, & Heiles 2006) or high-mass star-forming regions (Reid *et al*. 2019). However, the results are not spectacular, most probably because Classical Cepheids are not good tracers of the spiral structure. Most importantly, they are not as young as high-mass star-forming regions, and so could have moved far enough from their birthplaces and currently reside in a different arm or an inter-arm region (the rotational velocity of stars in the Galaxy differs from the speed of the spiral pattern, and depending on the distance from the Galactic center, stars are either faster or slower than the spiral pattern). Another uncertainty comes from the distance determination itself, as already shown in Figure 8, where distances determined by different groups differ by 2 – 3 kpc in the highly extincted regions toward the Galactic center.

On the other hand, as we clearly see in Figure 9, Cepheids do form some overdensities which resemble the spiral arms. It is however important to realize that this does not have to be the same spiral structure as represented by the youngest tracers such as neutral hydrogen or high-mass star-forming regions. Lemasle *et al*. (2022) showed that Classical Cepheids form segments of the spiral arms rather than long spiral arms, and some segments are consistent with the spiral structure formed by the youngest tracers. Interestingly, the locations of these segments seem to have little dependence on the age cut used to select Cepheids (see their Figures 8, D1, D2, and D3).

When looking at Figure 9, we see a paucity of stars on the far side of the Galaxy. Cepheids residing in the area behind the Galactic center cannot be discovered by optical surveys, because of extremely high extinction, but can be found by IR surveys which see through the dust (Dékány *et al*. 2015, 2019; Minniti *et al*. 2020). The caveat is that IR light curves of Cepheids are less unique and often resemble other types of variable stars, making the unambiguous classification impossible. For example, Cepheids from the VVV IR data released by Dékány *et al*. (2019), that had counterparts in the OGLE optical data, were verified by Skowron *et al*. (2019a). It turned out that only about 55% were genuine Cepheids, whereas the remaining ones were eclipsing binaries or spotted stars.

However, in their recent study, Minniti *et al*. (2020) classified IR candidates for Classical Cepheids using high-quality IR spectra, from which they were able to derive radial velocities and metallicities, that allow to separate Classical Cepheids from other stars without relying solely on their IR light curves. Even though the sample had only 30 stars, it allowed Minniti *et al*. (2021) to investigate the structure of the far side of the Milky Way disk. The authors showed that there is no significant warping of the far side of the disk within 12 kpc from the Galactic center, and that their data are consistent with the warped structure observed by Skowron *et al*. (2019a,b).

#### 3.2.2 The disk in Miras

Mira-type stars have been used as probes of Milky Way structure for over a century, but large catalogs of Miras became available only very recently (Iwanek *et al*. 2022). The first detailed three-dimensional map of the Galactic disk using Miras as tracers was presented by Iwanek *et al*. (2023), who showed that the disk seen in Miras is flared and possibly warped (see their Figure 7), although the latter is not as certain as in the case of Classical Cepheids, making it difficult to compare the degree of warping among different tracers. In their work on the Milky Way structure, Lemasle *et al*. (2022) showed that the degree of warping depends on the age of the tracer used, such that the older the tracer, the larger the degree of warping (see their Figure 3), meaning that the mean warp observed with Miras would be smaller that that of Classical Cepheids.

## 4 Summary

Period-luminosity relations are a powerful tool that can be used to study structures of galaxies, and in particular of our own Galaxy. So far, four groups of pulsating variable stars in the Milky Was have been successfully used to study the bulge, the disk, and the halo of the Milky Way: RR Lyrae-type stars, Classical Cepheids, *δ* Scuti stars, and Miras. These groups of stars are marked with red frames in Figure 1.

With the release of variable star catalogs from long-term surveys, it may seem that we have already discovered the majority of classical pulsators in the Milky Way, or at least, that the new discoveries will not significantly change the picture of our galaxy that had emerged from the analysis of the three-dimensional distribution of these variable stars. And while this may be true in the case of Classical Cepheids or RR Lyrae-type stars, there is still an unexplored wealth of long-period variables, among which only Miras have been partially examined so far. Beside Miras, long-period variables include OSARGs, SRVs, LSPs, eclipsing, and ellipsoidal variables (Wood *et al*. 1999; Soszyński *et al*. 2009, 2011b, 2013), all of which obey distinct period-luminosity relations (Figure 1), and may well serve as tracers of the Milky Way structure.

## Discussion

### Question (Ripepi)

I’m the one that calculated the distance in the Gaia Collaboration *et al*. (2023) paper. Just wanted to ask how much larger would the difference in the Wesenheit index have to be to explain such a large difference in distances (Figure 8).

### Answer

I didn’t check this, but that’s something that could be simulated. The differences in the Wesenheit index constant in the central regions of the Galaxy, as shown by Nataf *et al*. (2016), can vary from 1.5-1.6-1.9, so it would be good if someone checked how this translates to the distance difference.

### Question (Eyer)

I’m a bit surprised by your result about the Cepheid not tracing structures because we did a citizen science project where we asked people to classify objects and we have a projection of the Milky Way – the very nice picture done by people from NASA, it’s very nice and I was finding always there is the Cepheid: you see that in the Galactic plane and you see it’s near the arm, so that’s the Cepheid.

### Answer

Yes, that’s the thing with Cepheids. I don’t want to say that they don’t trace the spiral structure – they do trace some structures. And thank you for this question, because now I can show you a movie†. This is the movie where we simulated star forming episodes in the Galaxy in different spiral arms at different moments; the younger stars here are blue and the older ones are red. In the end, the simulation is compared with observations. This is a toy model, where stars move according to the rotation curve and have random motions of about 8 km/s, and the spiral pattern moves with a fixed velocity. You will see stars forming in one arm, but as the galaxy rotates, some of them actually move to the neighboring arm just because of the different velocity of the spiral pattern and of the stars. Some end up in the inter-arm region. So the answer is that Cepheids do trace a structure but it’s not necessarily consistent with the structure traced by younger traces, e.g. high-mass star-forming regions.

### Question (Anderson)

Did the preliminary comparison of Cepheid distances from different studies towards the bulge (Figure 8) consider information on color excess, e.g., *E*(*B* – *V*), to test whether the increased scatter indeed can be traced to the inadequacies of the reddening law?

### Answer

No, there is no sophisticated analysis here – just this simple comparison plot. But this is definitely something I will want to look into in more detail. We are at the point where we claim to have distances calculated with an accuracy better than 5% and we work on fine-tuning the period-luminosity relations. But in fact, the differences between distances calculated by different studies are much larger than that, which means that we really have to worry about other factors, for example about the reddening law.

### Comment (Hajdu)

About the extinction law implied by the Gaia Collaboration *et al*. (2023) results, the problem is that those are completely artificial and stem from incorrect zero point calibration in the VVV data that was done as a mistake by whoever did the calibration. It is shown both in my PhD thesis and in Hajdu *et al*. (2020). I told David Nataf that his results are completely artificial, and he agreed. You should not show this plot.

### Answer

I wasn’t aware of that, but maybe you’re right.

### Comment (Hajdu)

It’s a really unfortunate situation because it’s not his fault that his results are incorrect.

### Answer

It is definitely something to talk to David Nataf about, but it doesn’t change what we see on the plot. So even if we acknowledge that David Nataf’s work is wrong, it doesn’t change the fact that there is a correlation with extinction.

### Question (Riess)

So the difference plot you were showing is all with near-infrared data (Figure 8)?

### Answer

One comparison is between distances based on mid-infrared data and period-luminosity relations, in both studies. The other is between distances from a study based on mid-infrared and from a study based based on *Gaia* data and period-Wesenheit relations, so not infrared.

### Comment (Riess)

I don’t understand how it could be a cause of large extinction. Or it could be extinction if both the results use either near- or mid-infrared.

### Answer

Yes. So the blue plot is showing the comparison between studies where midinfrared was used, but there was a different treatment of extinction. The red plot compares studies where one used mid-infrared and the other used *Gaia*. And I don’t have an answer why.

### Comment(Riess)

OK, seems like more digging is required.

### Answer

Yes, more checking is needed. I’m going over each step of distance calculations to check where we start getting these differences. Definitely not in the periods – I already checked those and they are the same in all studies.

## Footnotes

†
http://www.astrouw.edu.pl/ogle/ogle4/OCVS/allGalCep.listID

†
http://www.astrouw.edu.pl/~jskowron/ogle/PR/milky_way_cepheids/cepheids_born_720.html
